# Cuproptosis: potential new direction in diabetes research and treatment

**DOI:** 10.3389/fendo.2024.1344729

**Published:** 2024-06-05

**Authors:** Jiashu Qu, Yifan Wang, Qiuyue Wang

**Affiliations:** Department of Endocrinology and Metabolism, The First Hospital of China Medical University, Shenyang, Liaoning, China

**Keywords:** copper, cell death, mitochondria, pancreatic β cells, type 2 diabetes

## Abstract

Cuproptosis, a recently discovered form of cell death, stems from an overabundance of copper ions infiltrating mitochondria. These ions directly engage lipoylated proteins, prompting their oligomerization and subsequent loss of iron-sulfur clusters. This sequence induces proteotoxic stress, ultimately culminating in cell death. Type 2 diabetes, a chronic metabolic disorder resulting from a complex interplay of genetic and environmental factors, has not yet been fully understood in terms of its etiology and pathogenesis. Intricately, it is linked to various modalities of cell death, including mitochondrial autophagy, apoptosis, pyroptosis, and ferroptosis. Studies have discovered impaired copper metabolism in individuals with Type 2 diabetes, hinting at a unique role for copper homeostasis in the progression of the disease. To this end, the present research aims to delineate the potential correlation between cuproptosis and Type 2 diabetes by exhaustively reviewing the existing literature. By synthesizing relevant research on cuproptosis, the paper intends to lay the groundwork for a thorough exploration of the pathogenesis of Type 2 diabetes and the development of targeted therapeutic interventions. The ultimate objective is to facilitate a deeper understanding of Type 2 diabetes and to identify novel therapeutic strategies associated with cuproptosis.

## 1 Introduction

Copper stands out as an essential micronutrient for most organisms, including humans, indispensable for fundamental biological processes (1). Functioning as a metallic element, it is tightly regulated in terms of absorption, distribution, and elimination within cells to sustain relatively low levels. This regulation is upheld by a sophisticated network of copper-dependent proteins, including cuproenzymes, copper chaperones, and membrane transport proteins. Together, they coordinate the influx, efflux, and intracellular utilization of copper ions, ensuring cellular copper levels remain within a specific range. This meticulous control mechanism safeguards against copper overload (2). Excess copper ions have long been known to induce cell death, but precise mechanisms have remained largely elusive until the introduction of cuproptosis. As a new form of cell death, cuproptosis was first discovered and experimentally confirmed by the research team led by Tsvetkov P (3). Cuproptosis, characterized as a form of programmed cell death, distinguishes itself from known modalities such as apoptosis, necroptosis, pyroptosis, and ferroptosis. Its primary mechanism involves the excessive influx of copper ions into mitochondria. Within mitochondria, they bind directly with lipoylated proteins, leading to the oligomerization of lipoylated proteins and subsequent loss of iron-sulfur clusters. This cascade ends in proteotoxic stress and eventual cell death.

Lipoylation is a specialized and highly conserved post-translational modification where lipoic acid is covalently attached to lysine residues in the lipoyl-binding domain of proteins, primarily through a thioester bond (4). In the human body, only four types of proteins undergo lipoylation, including ①Dihydrolipoyl transacetylase (DLAT), the E2 subunit of the pyruvate dehydrogenase complex, ②Dihydrolipoamide S-Succinyltransferase (DLST) – a crucial component of the α-ketoglutarate dehydrogenase complex (KGDC), ③Glycine Cleavage system protein H (GCSH) – an indispensable component of the Glycine Cleavage System (GCS), and ④Dihydrolipoamide branched chain transacylase E2 (DBT) – an essential part of the branched-chain α-ketoacid dehydrogenase complex (BCKDC). All these proteins are localized within mitochondria, and actively participate in the tricarboxylic acid cycle, playing a pivotal role in maintaining the homeostasis of mitochondrial function. Consequently, aberrant binding of excessive copper ions to these proteins disrupts their functionality, leading to compromised mitochondrial function. Iron-sulfur proteins feature the presence of iron-sulfur clusters (Fe-S clusters) (5) and exert a significant influence within the mitochondrial oxidative respiratory chain, spanning complexes I, II, III and several other mitochondrial enzymes. In this case, the loss of iron-sulfur proteins resulting from cuproptosis impairs the integrity of the mitochondrial oxidative system, eventually culminating in cell death.

**Figure 1:**
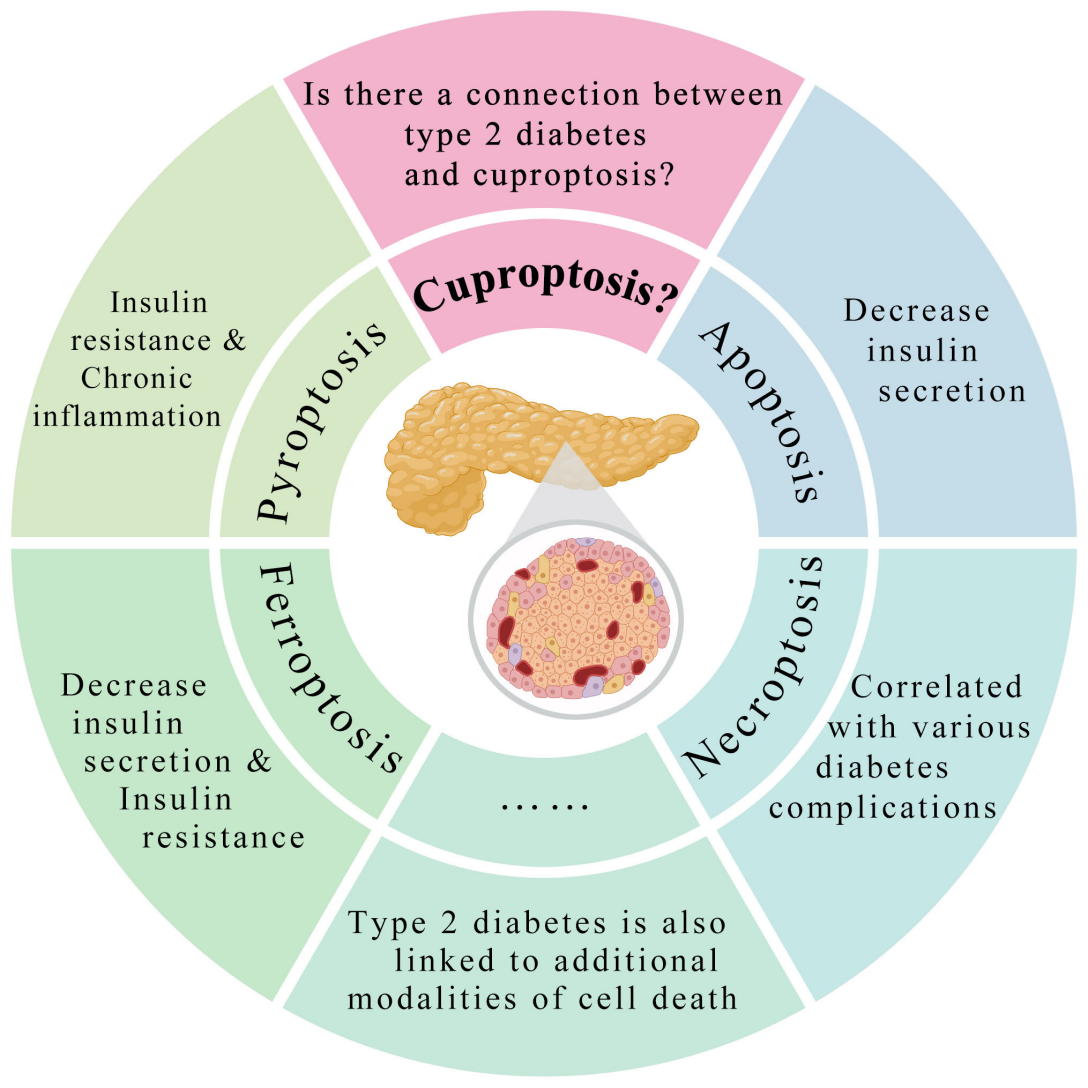
Type 2 diabetes is intricately linked to various modalities of cell death. Type 2 diabetes is intricately linked to various modalities of cell death, including apoptosis, necroptosis, pyroptosis, and ferroptosis. However, it remains to be explored whether there a connection between Type 2 diabetes and cuproptosis.

Type 2 diabetes is a chronic metabolic disorder arising from the intricate interplay of genetic and environmental factors, anticipated to affect 693 million adults worldwide by 2045. Its pathophysiological hallmark lies in dysfunction in pancreatic β cells, insulin resistance, or a concomitant combination of both, resulting in hyperglycemia attributable to absolute or relative insulin deficiency (57). However, the complete etiology and pathogenesis of Type 2 diabetes remain incompletely elucidated. The impairment of pancreatic β cell function in patients with Type 2 diabetes is closely correlated with cell death, with compelling evidence linking it with various forms of programmed cell death, including mitochondrial autophagy, apoptosis (58), pyroptosis (59) and ferroptosis (60) (**Figure 1**). In addition, extensive investigations into the intricate association between Type 2 diabetes and copper have been recently conducted (61, 62). Signs of systemic copper overload in patients with Type 2 diabetes and animal models include heightened urinary copper excretion, increased copper balance, normal or elevated plasma copper ion and ceruloplasmin levels, and notably elevated levels of copper ions in the liver and kidneys (25). Meta-analyses have also pointed out elevated serum copper ion levels in patients with Type 2 diabetes compared to the healthy control group (63). It should be noted that Chang W et al. extensively reviewed the literature on the relationship between dietary copper/plasma copper and diabetes in clinical studies from 2019 to 2023 (64). Multiple studies concerning the dietary copper/plasma copper levels in patients with diabetes were summarized. However, these studies have presented inconsistent conclusions, suggesting more complex relationship between copper and diabetes, which may be poorly understood. Nevertheless, this article clearly and affirmatively underscores the positive significance of copper chelators in the treatment of diabetes. These studies indicate that diabetes may impair copper metabolism in patients, with defects in copper regulation potentially influencing the pathogenesis and progression of the disease. Therefore, investigating the balance of copper metabolism in diabetes regulation offers a novel direction for understanding the mechanisms underlying diabetes.Consequently, based on the groundbreaking discoveries regarding cuproptosis by the research team led by Tsvetkov P (3), genes including LIAS, FDX1, LIPT1, DLD, DLAT, PDHA1, PDHB, MTF1, GLS, CDKN2A, SLC31A1, ATP7A, and ATP7B, along with the associated proteins Glutathione and HSP70 mentioned in this article related to cuproptosis, were gathered. Literature databases were searched for studies linking diabetes with these genes or proteins (**Table 1**). Ultimately, the collected literature was hereby summarized to elucidate the potential relationship between cuproptosis and diabetes and to explore novel therapeutic avenues for diabetes.

**Table 1:** Genes or proteins related to cuproptosis mentioned in this article.

Name	Type	Function	Role in cuproptosis	Role in diabetes
DLAT	Protein-coding gene	E2 constituent of PDC (6, 7)	The oligomerization of DLAT can result in the deactivation of PDC (3, 8)	Maintain the morphology and functionality of pancreatic β cells (9, 10)
DLD	Protein-coding gene	E3 constituent of PDC (11)	No mention	Reduce protein abundance in the visceral adipose tissue of patients with T2DM (12)
PDHA1	Protein-coding gene	E1 constituent of PDC (13)	No mention	Maintain the morphology and functionality of pancreatic β cells (14)
PDHB	Protein-coding gene	E1 constituent of PDC (13)	No mention	Maintain the morphology and functionality of pancreatic β cells (15)
PDC	Protein complex	Convert pyruvate to acetyl-CoA (16, 17)	The deactivation of PDC leads to malfunctions in the TCA and mitochondrial functions (3)	Maintain the morphology and functionality of pancreatic β cells, participates in insulin secretion (9, 10, 18–20)
SLC31A1	Protein-coding gene	Enabling selective copper uptake (21–24)	Overactivation lead to intracellular copper accumulation (3)	Be highly associated with diabetic cardiomyopathy (25, 26)
ATP7A	Protein-coding gene	Copper efflux protein (27)	Knock out lead to intracellular copper accumulation (3)	Decreased in patients with diabetes (28, 29)
ATP7B	Protein-coding gene	Copper efflux protein (27)	Knock out lead to intracellular copper accumulation (3)	Insulin can promote the return of ATP7B to the Golgi apparatus, thereby aiding in the clearance of excess copper ions from the cell (30, 31)
CDKN2A	Protein-coding gene	Encode p16 and p14ARF; Regulate the cell cycle (32, 33)	Reduce the sensitivity of cells to copper ionophores (3)	CDKN2A is significantly associated with susceptibility to T2DM; CDKN2A can lead to premature aging of pancreatic islets and insulin resistance (34, 35)
HSP70	Protein family	Shield cells from stress (36–38)	Cellular cytotoxic stress marker induced by cuproptosis (3)	eHSP70 is related to insulin resistance; iHSP70 is related to pancreatic beta cell injury (39–46)
Glutathione	Tripeptide	Antioxidant and cytoprotectant (47, 48)	As an endogenous copper chelator, inhibiting cuproptosis (3)	Glutathione is decreased in patients with diabetes (49–56)

## 2 Materials and methods

### 2.1 Search strategy

After systematic literature search from PubMed (https://pubmed.ncbi.nlm.nih.gov), Google Scholar (https://scholar.google.com), and Web of Science (https://www.webofscience.com) databases until December 2023, the relationship between the genes or proteins related to cuproptosis as discussed in the aforementioned Tsvetkov P article and diabetes was investigated. The search was conducted using the search terms “LIAS”, “FDX1”, “LIPT1”, “DLD”, “DLAT”, “PDHA1”, “PDHB”, “MTF1”, “GLS”, “CDKN2A”, “SLC31A1”, “ATP7A”, “ATP7B”, “Glutathione”, “HSP70” and “Diabetes Mellitus”[MeSH]. Relevant articles were selected by classifying the title, abstract, and full text of all the studies and limiting the included studies to those published in English. Two authors, Jiashu Qu and Yifan Wang, selected the articles independently, and reviewed the abstracts and full text of the articles.

### 2.2 Inclusion and exclusion criteria

Studies were considered eligible if satisfying all the following criteria: ①the content of the article focused on diabetes and one or more genes or proteins related to cuproptosis; ②*in vitro* studies, animal studies, clinical studies, case reports, or reviews that meet criterion ①; and ③written in English. In contrast, the exclusion criteria included: ①not written in English; ②not peer-reviewed or formally published literature; or ③theses or conference papers. The selection process was independently completed by two authors, and differences were resolved through consultation or discussion with the third author.

## 3 Potential link between cuproptosis and diabetes

Before the concept of cuproptosis emerged, attention had already been drawn to the impact of copper on pancreatic β cells in diabetes. Focus centered on how copper contributed to the aggregation of human islet amyloid polypeptide (hIAPP) and the formation of cytotoxic oligomers (65). These copper-hIAPP complexes possess metallopeptide complex structures with a low aggregation potential and may give rise to granular oligomers serving as the primary drivers of increased copper-mediated hIAPP cytotoxicity (66). It is anticipated that these granular oligomers will exhibit characteristics typical of toxic oligomers, inducing membrane destabilization and ultimately resulting in cell apoptosis (67). Furthermore, copper-promoted generation of reactive oxygen species (ROS), such as H2O2, and disruption of mitochondria also impact the degeneration of islet cells, thereby promoting apoptosis (68). The present study aims to draw attention to the expression and roles of cuproptosis-related genes or proteins in diabetes, so as to provide an opportunity to re-examine the impact of copper on Type 2 diabetes mellitus and pancreatic β cells. This offers a novel perspective for gaining a deeper understanding of diabetes pathogenesis.

### 3.1 PDC and its components including DLAT, DLD, PDHA1 and PDHB

The pyruvate dehydrogenase complex (PDC) is a crucial enzyme assembly bridging glycolysis and the tricarboxylic acid cycle (TCA), catalyzing the conversion of pyruvate, a product of glycolysis, into acetyl-CoA. This reaction serves as a primary gateway for carbohydrates to enter the citric acid cycle, a fundamental process ubiquitous across all biological systems (16). In eukaryotes, the PDC consists primarily of three enzymes, which are also referred to as the three subunits of PDC. Additionally, it includes five auxiliary factors, forming the fundamental structure of the PDC. The three enzymes are pyruvate dehydrogenase, dihydrolipoyl transacetylase (DLAT), and dihydrolipoamide dehydrogenase (DLD). Pyruvate decarboxylase constitutes the E1 subunit of PDC, primarily composed of Pyruvate dehydrogenase alpha 1 (PDHA1) and Pyruvate dehydrogenase beta (PDHB). DLAT and DLD form the E2 and E3 subunits of PDC, respectively. Additionally, the five auxiliary factors constituting PDC are thiamin pyrophosphate (TPP), nicotinamide adenine dinucleotide (NAD), flavin adenine dinucleotide (FAD), lipoic acid, and CoA (17). As a complex enzyme assembly, any aberration or deficiency in any component would render PDC incapable of performing its normal function.

Tsvetkov P pointed out that DLAT, PDHA1 and PDHB, among genes involved in cuproptosis, are indeed key genes encoding the constituents of PDC (3). DLAT is located on human chromosome 11q23.1, encoding the E2 subunit of PDC. As previously mentioned, DLAT is one of the four proteins in the human body capable of undergoing lipoylation. Within DLAT lies the lipoic acid domain. Lipoic acid, under the catalysis of lipoic acid synthetase (LIAS), forms a covalent bond with the lysine residues in the lipoic acid domain of DLAT in the form of an amide bond, giving rise to its lipoylation. Due to lipoamide’s larger size compared to DLAT, this modification not only impacts protein structure but also facilitates a “swinging arm” function during enzyme-catalyzed reactions. Consequently, the lipoyl swinging arms on DLAT (E2) interact with the E1 and E3 subunits on the exterior of PDC, catalyzing the decarboxylation of pyruvate and the acyl activation of coenzyme A (6, 7). In the mechanism of cuproptosis, excess copper binds to lipoylated proteins, leading to oligomerization of lipoylated proteins and subsequent loss of function. This process leads to aggregation and inactivation of DLAT. Notably, Gao F et al. proposed DLAT as a promoter of cuproptosis (8). In their study of cuproptosis, the research team led by Tsvetkov P utilized genome-wide CRISPR-Cas9 loss-of-function screens, and found that, apart from DLAT, positive hits were also yielded in DLD, PDHA1 and PDHB in the PDC (3). DLD is located on human chromosome 7q31.1, encoding the components of the E3 subunit of PDC (11). PDHA1 and PDHB are on human chromosome Xp22.12 and chromosome 3p14.3, respectively, encoding two components of the E1 subunit of PDC, PDHA1 and PDHB. The combination of two PDHA1 and two PDHB serves as a tetramer to form the primary structure of the E1 subunit (13). Although Tsvetkov P did not specify the roles of DLD, PDHA1 and PDHB in cuproptosis, the entire PDC, including DLD, PDHA1 and PDHB, tended to fail to function normally due to the complexity and close association of PDC as well as the inactivation of DLAT caused by oligomerization. PDC functions as a bridge between glycolysis and the tricarboxylic acid cycle, participating in energy production, metabolic regulation, and maintenance of cellular respiration. Therefore, abnormal functioning of PDC deals a significant blow to the normal operation of mitochondria and cells.

PDC and its constituents play a pivotal role in the onset and progression of diabetes. PDC catalyzes the conversion of pyruvate to acetyl-CoA, facilitating its involvement in the TCA cycle. The ATP/ADP ratio is increased, leading to the closure of plasma membrane ATP-dependent K^+^ channels. Voltage-gated Ca^2+^ channels open, triggering insulin exocytosis. Amplification of metabolic coupling factors enhances insulin secretion, demonstrating PDC as a crucial regulatory component in modulating insulin exocytosis (9). Therefore, loss of PDC activity results in disrupted glucose metabolism and cellular dysfunction, affecting β-cell function and insulin release. Additionally, numerous animal experiments have confirmed significantly reduced PDC activity in diabetic animal models (10). In clinical studies, Lars W. Andersen et al. observed decreased PDC activity in DKA patients with diabetes (18). Indeed, PDC is not only associated with diabetes but also closely linked to its complications. Extensive research has explored PDC in diabetic cardiomyopathy, where decreased PDC activity in cardiomyocytes stands out as a pivotal pathological alteration (19, 20).

A proteomic study has indicated reduced protein abundance of DLD in the visceral adipose tissue of patients with T2DM (12). The role of the constituent PDHA1 in diabetes is also being gradually elucidated. XIAO W et al. constructed a PDHA1 gene knockout mouse (βKO) model by knocking out the PDHA1 gene in mice, and found that βKO mice exhibited impaired glucose tolerance and significantly reduced pancreatic islet secretion function, without presenting impaired insulin sensitivity. This suggested the correlation between the cause of hyperglycemia in βKO mice and impaired pancreatic β-cell function and insulin secretion dysfunction rather than peripheral tissue insulin resistance. The finding further indicated the essential role of PDHA1 in pancreatic islet development, and its absence significantly impacted the morphology and function of pancreatic islet cells (69). Furthermore, research has also suggested that T cells may mediate tubular injury in diabetic nephropathy via impaired PDHA1 and autophagy in Type 1 Diabetes (14). PDHB has garnered widespread attention as a novel diabetes risk gene (15), but further research is still needed to elucidate its role in diabetes.

In conclusion, there is a growing recognition of the impact of PDC and its constituent components, DLAT, DLD, PDHA1, and PDHB, on diabetes. The unveiling of cuproptosis has introduced fresh avenues for investigating how PDC and its constituent parts impact the development and advancement of diabetes from this innovative perspective. This offers a promising research direction with significant potential for further exploration.

### 3.2 SLC31A1

SLC31A1 is located at 9q32 on human chromosome 9, encoding solute carrier family 31 member 1. SLC31A1 serves as the primary regulatory factor for copper uptake and is extensively expressed within cells (21). Alterations in SLC31A1 expression levels directly impact levels of intracellular copper ion. As an integral membrane protein localized to the plasma membrane and endosomal vesicles, SLC31A1 plays a broad and distinctive role in copper absorption. SLC31A1 presents as a trimeric ion channel-like structure, selectively ferrying copper into the cell through a central pore within the trimer (22). Copper in the human body is obtained primarily through dietary intake, particularly from organ meat and shellfish, and is absorbed through SLC31A1 in the epithelial cells of the small intestine (23). Cellular copper uptake largely relies on the abundance of SLC31A1 on the cell membrane, a process regulated in response to copper levels. When extracellular copper levels rise, SLC31A1 is rapidly internalized from the cell membrane via endocytosis, thereby reducing copper uptake and safeguarding cells against excessive copper accumulation. In contrast, in the case of reduced levels of extracellular copper, SLC31A1 returns to the cell membrane to resume its copper uptake function. Through a negative feedback regulatory mechanism, SLC31A1 strictly controls intracellular copper levels, maintaining cellular copper homeostasis (24).

The negative feedback regulation mechanism of SLC31A1 in normal cells helps prevent copper overload. Therefore, in the study of cuproptosis, Tsvetkov P utilized copper ionophores like Elesclomol to introduce copper into cells. Although how the complex of Elesclomol and copper enters the cell currently remains unclear, it still functions well in delivering copper to mitochondria (70). Tsvetkov P claimed that the method of increasing intracellular copper levels using copper ionophores shared similarities with diseases caused by SLC31A1 abnormalities leading to an imbalance in copper homeostasis. They overexpressed SLC31A1 in cells, significantly increasing the sensitivity of cells to physiological copper concentrations. Furthermore, these cells overexpressing SLC31A1 exhibited indicators associated with cuproptosis, similar to normal cells treated with copper ionophores. Meanwhile, the use of copper chelators effectively saved their death (3).

However, research on SLC31A1 in Type 2 diabetes is still in its early stages. Huo S found that an excess of copper in patients with diabetes upregulated SLC31A1, leading to functional damage in cardiomyocytes in diabetic cardiomyopathy, which was possibly related to cuproptosis (26). Additionally, Zhang S found that after 10 weeks of STZ induction in diabetic rats, there presented a significant deficiency in left ventricular copper levels, and the expression of SLC31A1 was significantly reduced. Treatment of diabetic rats with the copper chelator triethylenetetramine (TETA) fully restored left ventricular copper levels to normal while significantly improving heart function. However, SLC31A1 expression was not restored (25).

SLC31A1 serves as a crucial channel for copper uptake, contributing significantly to maintaining copper homeostasis within the cell. While the occurrence of diabetic cardiomyopathy is indeed linked to abnormal expression of SLC31A1 in cardiac myocytes, it remains unclear whether similar abnormalities exist in pancreatic β cells. This knowledge gap warrants further exploration and investigation.

### 3.3 ATP7A and ATP7B

ATP7A is located on chromosome X at Xq21.1, while ATP7B is on chromosome 13 at 13q14.3. These two genes encode two distinct proteins, i.e., ATP7A and ATP7B, both of which are P-type copper-transporting ATPases, sharing 60% identity and functional homology. Meanwhile, each contains eight transmembrane domains, six cytoplasmic N-terminal domains rich in cysteine metal binding motifs (MXCXXC), a typical transmembrane CPX metal binding motif, as well as ATP binding and aspartic acid phosphorylation domains (27). ATP7A and ATP7B harness the energy produced by ATP hydrolysis to actively pump copper out of the cytoplasm, traversing the lipid bilayer. Although the expression of these two proteins varies in different cells and tissues, their main functions involve the distribution and efflux of copper within the cell, making ATP7A and ATP7B crucial copper efflux proteins. Wilson’s disease is a hereditary disorder of copper metabolism resulting from mutations in the ATP7B gene. These mutations disrupt copper homeostasis, leading to copper overload primarily in the liver, brain, and other organs (71).

SLC31A1 and ATP7A/ATP7B jointly maintain intracellular copper homeostasis. Similar to SLC31A1, the method to increase intracellular copper levels using copper ionophores, as proposed by Tsvetkov P, shares commonalities with diseases resulting from abnormalities in ATP7A/ATP7B, leading to unbalanced copper homeostasis. Tsvetkov P and his team compared mice lacking the ATP7B gene (a mouse model for Wilson’s disease) with normal mice, and observed typical indicators of cuproptosis in liver cells of mice lacking the ATP7B gene, including lipoylated protein oligomerization, depletion of iron-sulfur proteins, and increased abundance of HSP70 (3).

Both ATP7A and ATP7B are highly relevant to diabetes. Hil´ario-Souza found that insulin could regulate ATP7B activity, promoting its return to the Golgi apparatus. This insulin regulation facilitates to remove copper from cells and maintain intracellular copper homeostasis (30). The present research was piqued by the case of a patient suffering from Wilson’s disease with severe diabetes. As reported by Li J, treatment with copper chelators such as D-penicillamine resulted in improved pancreatic function in patients, evidenced by HbA1c levels approaching normal and a reduction in insulin usage. This is the first report of the onset of diabetes caused by Wilson’s disease. Li J believed that the improvement observed was likely attributed to ATP7B and copper overload-induced pancreatic β cell death (31). Notably, Sudhahar V observed a significantly reduced expression of ATP7A in the vasculature of diabetic mice (28), and a decrease in ATP7A expression in extracellular vesicles of patients with diabetes and animal models was discovered by Abdelsaid K, which could be partially restored through exercise (29).

ATP7A and ATP7B matter considerably in the efflux of copper from cells and in the maintenance of copper homeostasis. These cases mentioned above collectively suggest that ATP7A and ATP7B could be involved in the onset and progression of diabetes. Further investigation of the deep links between ATP7A/ATP7B and diabetes should be conducted to provide a deeper understanding of the development of this condition.

### 3.4 CDKN2A

Cyclin-dependent kinase inhibitor 2A (CDKN2A) is located on human chromosome 9p21, encoding two important proteins, i.e., p16 and p14ARF, which regulate the cell cycle. The progression of the cell cycle is regulated by four key checkpoints including the G1-S transition, the S-phase checkpoint, the G2-to-M transition, and the mitotic spindle checkpoint (32). The expression of p16 can prevent cells from transitioning from G1 to S, thereby leading to cell cycle arrest (33). This role of p16 has garnered widespread attention and triggered research in the fields of cancer and cellular senescence (72).

While investigating cuproptosis, Tsvetkov P identified CDKN2A as a negative regulator of copper ion carrier sensitivity through a Whole-genome CRISPR-Cas9 positive selection screen (3). In other words, the expression of CDKN2A could inhibit the sensitivity of cells to copper ion carriers, thereby partially inhibiting or preventing cuproptosis. Unfortunately, Tsvetkov P did not extensively study and elucidate how CDKN2A negatively regulated the sensitivity of cells to copper ion carriers. The important and complex regulatory role of CDKN2A in the cell cycle necessitates further research on the role of CDKN2A in cuproptosis.

Type 2 diabetes, as a complex metabolic disease influenced by multiple genes, has drawn considerable attention regarding the role of CDKN2A as a risk gene. Within the Asian population (73), the correlation between CDKN2A and Type 2 diabetes has been extensively studied in various countries and regions, including but not limited to Japan (74), China (75–77), India (78), and Thailand (79). Genome-Wide Association Study (GWAS) is generally utilized to compare Single Nucleotide Polymorphisms (SNPs) of the CDKN2A gene between patients with Type 2 diabetes and normal control groups. All these studies have demonstrated the significant correlation between the CDKN2A gene and susceptibility to Type 2 diabetes. Besides, it is worth mentioning that CDKN2A is not only associated with Type 2 diabetes, but also with diabetic nephropathy (80) and gestational diabetes (81). CDKN2A can impact the occurrence and development of Type 2 diabetes through multiple mechanisms. Abnormal expression of CDKN2A, a gene involved in regulating the cell cycle, can lead to premature aging of islets, contributing to dysfunction of pancreatic β-cells and inadequate insulin secretion (34). Additionally, abnormal expression of CDKN2A in adipose tissue may lead to insulin resistance (35).

In summary, CDKN2A, a crucial gene regulating the cell cycle, holds significance in diabetes. However, its specific role in cuproptosis remains incompletely understood. Therefore, further research is needed to uncover the involvement of CDKN2A in cuproptosis and explore potential connections between CDKN2A and the regulation of both cuproptosis and diabetes.

### 3.5 HSP70

HSP70 (70kDa heat shock proteins) is a highly conserved molecular chaperone expressed in almost all biological cells, which involves three major functional domains, i.e., the N-terminal ATPase domain, the substrate binding domain, and the C-terminal domain (36). HSP70 can prevent aggregation and promote the refolding of misfolded denatured proteins, solubilize aggregated proteins, and cooperate with cellular degradation machinery to clear aberrant proteins and protein aggregates (37). These actions get HSP70 significantly involved in protecting cells and maintaining normal cell functions and metabolism. Moreover, HSP70 holds considerable significance in mitigating proteotoxic stress within cells. Proteotoxic stress arises from the accumulation or misfolding of proteins, which can stem from various cellular disturbances or insults (82). Acting as a key player in this scenario, HSP70 prevents protein aggregation, facilitates the refolding of misfolded proteins, and assists in the clearance of aberrant protein aggregates (83). This function is particularly important in maintaining cellular homeostasis and preventing cellular damage. By responding to a wide range of stressors, both internal and external, HSP70 effectively alleviates proteotoxic stress, thus safeguarding normal cell functions and metabolism. HSP70 is indeed categorized as a stress protein due to its induction by various stressors. As such, it serves as an effective buffering system against cellular stress, whether triggered by external factors like physiological, viral, or environmental stressors, or internal factors such as replicative or oncogenic stimuli (38).

During the cuproptosis process, excessive copper enters the cell mitochondria, leading to the oligomerization of lipoylated proteins and the loss of iron-sulfur proteins. Subsequently, the expression of HSP70 is induced, reflecting an acute proteotoxic stress within the cell. The elevated expression of HSP70 in cuproptosis is considered an indicator of evaluating cellular toxic stress. Tsvetkov P detected an increase in HSP70 abundance in both cells using copper ionophores and in the liver of ATP7B gene-deficient mice (a mouse model for Wilson’s disease) (3). Although HSP70 does not exert detrimental effects on cells and organisms, rather serving as a crucial protein that protects them from harmful environmental stressors, its increased expression means that cells and organisms are experiencing significant stress. Therefore, Tsvetkov P regarded HSP70 as a critical indicator of cellular exposure to cuproptosis.

HSP70 holds much attention in research regarding Type 2 diabetes. Recent findings underscore the importance of HSP70 as a central player in the pathophysiology of β-cell dysfunction, insulin resistance, and complications such as diabetic microangiopathy (39). In particular, HSP70 appears to serve a dual function, with intracellular HSP70 (iHSP70) exhibiting anti-inflammatory properties, while extracellular HSP70 (eHSP70) demonstrating a pro-inflammatory effect, contributing to both local and systemic inflammation (40). Multiple investigations have pointed to an up-regulation of eHSP70 expression in individuals affected by Type 2 diabetes (41). Binding of eHSP70 to various cell TLR4 receptors activates JNK, thereby unleashing a cascade of inflammatory mediators. This process promotes the onset of chronic inflammation in patients with diabetes (42). Furthermore, JNK activation significantly compromises insulin signal transduction, resulting in diminished tissue sensitivity to insulin as well as the emergence of insulin resistance (84). Consequently, while the precise origins of eHSP70 in patients with diabetes are not fully understood, elevated plasma levels of eHSP70 are closely correlated with insulin resistance and chronic inflammation in this population. The expression of iHSP70 in patients with diabetes is a matter of debate, with conflicting reports suggesting both elevated and reduced levels. Some perspectives suggest that hyperglycemia in diabetic patients may trigger increased iHSP70 expression, potentially leading to cellular damage (43). On the contrary, others posit that the decrease in iHSP70 reduces the protective function of pancreatic β-cells, further leading to their impairment and the eventual development of Type 2 diabetes (44). Given the asynchronous patterns of eHSP70 and iHSP70, Krause M and colleagues presented an intriguing concept concerning the chaperone balance hypothesis. This theory postulates that the ratio of extracellular to intracellular HSP70 content may dictate the outcome of inflammation and related insulin resistance (45). Based on this notion, Mulyani proposed targeted regulatory strategies for HSP70 aimed at improving insulin resistance in patients with diabetes and facilitating the effective management of the health of patients with Type 2 diabetes (46).

In summary, HSP70 serves as a critical indicator of cellular stress and a vital buffer for protecting cells during stress. In individuals with Type 2 diabetes, iHSP70 exhibits anti-inflammatory functions, while eHSP70 simultaneously promotes inflammation. Although Tsvetkov P considered HSP70 a vital indicator of the cellular response to cuproptosis, the involvement of other proteins within the HSP family in cuproptosis remains to be explored. Besides, the influence of cuproptosis on the expression of HSP70 in patients with Type 2 diabetes should be further clarified.

### 3.6 Glutathione

Glutathione, a tripeptide composed of glutamate, cysteine, and glycine, is pivotal in eliminating various active substances. It serves as a vital cellular and systemic reducing agent and primary antioxidant, rigorously controlling the redox state. I5t directly participates in antioxidant functions by scavenging reactive oxygen species (ROS) and reactive nitrogen species (RNS) and indirectly through glutathione-dependent peroxidase-catalyzed reactions (47). Furthermore, glutathione serves as a mediator in numerous other physiological responses, including cell signaling, xenobiotic metabolism, thiol disulfide exchange reactions, and serving as an essential cysteine reservoir (48). Cellular glutathione levels are remarkably sensitive to environmental factors, with various stress conditions such as heavy metals, high glucose concentrations, and heat shock capable of altering glutathione levels. Thus, glutathione plays a crucial role in facilitating detoxification reactions and effectively preventing cellular damage induced by oxidative stress.

Glutathione functions as an endogenous copper chelator (85). In cuproptosis research, Tsvetkov P observed increased expression of cuproptosis-related indicators upon the addition of buthionine sulfoximine (BSO) to cell culture medium. This effect arises from the depletion of endogenous glutathione, a copper chelator, subsequently compromising the protective mechanisms of cells and mitochondria. Consequently, disrupted glutathione levels make copper homeostasis more susceptible to imbalances and the appearance of cuproptosis. Hence, glutathione can be considered a protective element against cuproptosis or, alternatively, as a negative regulator of cuproptosis.

Glutathione depletion has indeed become a prevalent characteristic in numerous diseases, including Type 2 diabetes. The diminished expression and depletion of glutathione in the bodies of individuals with diabetes have been linked to several underlying mechanisms. Glutamate cysteine ligase (GCL), a crucial enzyme responsible for catalyzing the initial step of glutathione synthesis, has been observed to be expressed at reduced levels in the bodies of individuals with diabetes. This decrease in GCL expression directly affects glutathione synthesis, probably related to the observed increase in transforming growth factor β (TGF-β) levels in patients with Type 2 diabetes (49). TGF-β signaling plays various roles in the development, function, proliferation, apoptosis, and dedifferentiation of β cells (50). Furthermore, hyperglycemia has been shown to accelerate the enzymatic conversion of glucose to sorbitol by sorbitol dehydrogenase, accompanied by a reduction in NADPH and glutathione, thus increasing cell sensitivity to oxidative stress. Another notable source of reactive oxygen species (ROS) generation in β cells is the heightened demand for insulin, particularly evident in cases of insulin resistance. This increased demand prompts elevated hormone synthesis, contributing to ROS production (51). This phenomenon is a consequence of insulin processing that requires the formation of disulfide bonds, a process that generates a substantial amount of ROS, depletes the glutathione reservoir, and ultimately induces oxidative stress (52). The therapeutic potential of exogenous glutathione supplementation has been extensively explored for the treatment and management of Type 2 diabetes, orally or by injection. For instance, a randomized clinical trial revealed that a three-week oral glutathione supplementation regimen significantly improved systemic insulin sensitivity in patients with Type 2 diabetes (53). However, another clinical study showed that glutathione supplementation in adolescents with type 1 diabetes did not increase glutathione pool reserves or ameliorate oxidative stress (54). Conversely, Pimson’s animal experiments demonstrated that glutathione supplementation in Type 1 diabetes mice improved their antioxidant balance (55). Besides, notably, Karolczak K found that the glutathione concentration in the bodies of patients with diabetes appeared to be inversely correlated with fasting blood glucose levels. In other words, the poorer the control of fasting blood glucose in patients with diabetes, the lower the concentration of glutathione in their bodies (56).

In general, glutathione plays a crucial role as an antioxidant in protecting the body from oxidative stress. Its function as an endogenous copper chelator matters considerably in preserving cells during cuproptosis. In the context of diabetes, patients are more vulnerable to the impact of oxidative stress due to reduced glutathione levels, which further intensifies the progression of the disease. Although the precise connection between decreased glutathione levels in diabetes and cuproptosis remains unclear, the potential of glutathione as a protective agent presents significant avenues for further research.

## 4 Conclusions and future perspectives

**Figure 2:**
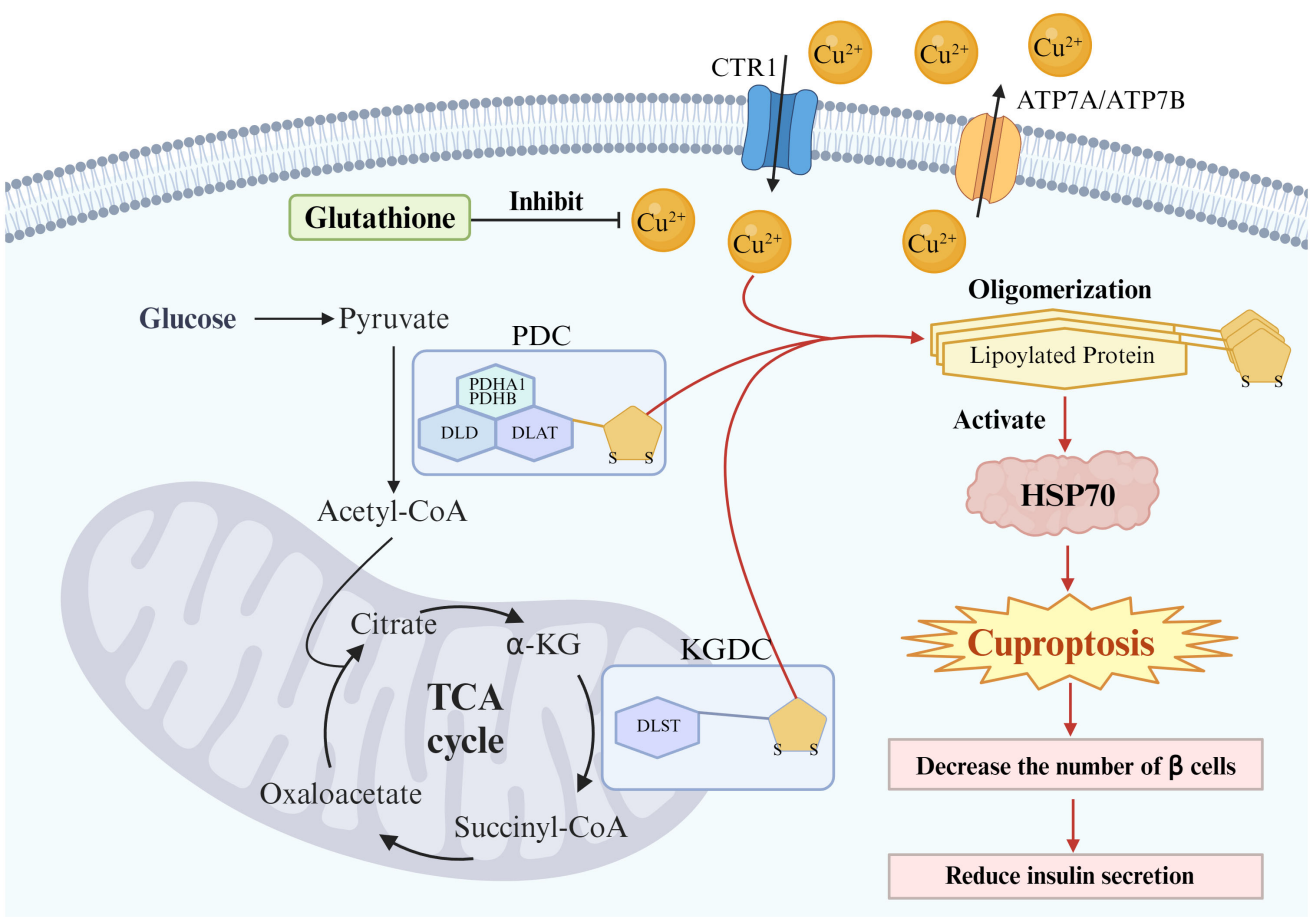
Potential links between cuproptosis and Type 2 diabetes in β cells. The primary mechanism of cuproptosis is attributed to the excessive influx of copper ions into mitochondria, where they bind directly with lipoylated proteins, leading to oligomerization of lipoylated proteins. This cascade culminates in proteotoxic stress (activation of HSP70) and eventual cell death. Pancreatic β cells may undergo cuproptosis, leading to a reduction in the number of these cells and subsequently decreasing insulin secretion. Glutathione serves as an endogenous copper chelator and protects Pancreatic β cells from cuproptosis.

Copper serves as an essential trace element for normal human growth and development, actively participating in various physiological processes within the organism. The maintenance of specific concentrations of copper in the body is integral to its homeostasis. However, an imbalance in copper homeostasis may lead to the occurrence and progression of diseases through the induction of cuproptosis, a recently proposed form of programmed cell death by Tsvetkov et al. In this review, mechanisms underlying copper-induced cell death were examined. The primary mechanism of cuproptosis is attributed to the excessive influx of copper ions into mitochondria, where they bind directly with lipoylated proteins, leading to the oligomerization of lipoylated proteins and subsequent loss of iron-sulfur clusters. Meanwhile, this cascade culminates in proteotoxic stress and eventual cell death. Besides, a comprehensive set of genes mentioned in this study related to cuproptosis were gathered, including LIAS, FDX1, LIPT1, DLD, DLAT, PDHA1, PDHB, MTF1, GLS, CDKN2A, SLC31A1, ATP7A, ATP7B, along with associated proteins Glutathione and HSP70. Through a literature search strategy in Section 2, the relationship between these genes or proteins and diabetes was also investigated. Specifically, roles and research progress of DLAT, DLD, PDHA1, PDHB, PDC, SLC31A1, ATP7A, ATP7B, HSP70, and Glutathione in cuproptosis and diabetes were extensively discussed. Generally, these genes and proteins exhibit consistent expression patterns in copper-induced cell death and diabetes, suggesting a potential pathological mechanism linking cuproptosis to diabetes. This hypothesis was further supported a recent bioinformatics study on the correlation between cuproptosis-related genes and diabetes immune infiltration (86). It should be noted that within the genes detailed, the roles of DLD, PDHA1, PDHB, CDKN2A, and others in cuproptosis were not explicitly elucidated by Tsvetkov et al., indicating directions for further investigation into cuproptosis. Additionally, due to a lack of relevant research in the field of diabetes, LIAS, FDX1, LIPT1, MTF1, GLS, among others were not involved, or the limited literature did not align with the search strategy adopted. In other words, studying these genes represents a potential avenue for further exploration. Overall, in this review, related studies of genes and proteins involved in both cuproptosis and diabetes were summarized for the first time the, hinting at a potential relationship between cuproptosis and diabetes from this perspective (**Figure 2**). It is expected that this review could provide a foundational knowledge framework and inspiration for research in this area, advancing further investigation and discussion on the relationship between copper-induced cell death and diabetes. the ultimate objective is to facilitate a deeper understanding of Type 2 diabetes and identify novel therapeutic strategies associated with cuproptosis. However, this research still faces significant challenges and a long road ahead. Firstly, further research into the mechanisms of cuproptosis, such as the destination of lost iron-sulfur clusters and the specific roles of some genes mentioned earlier should still be carried out. Additionally, the presence of other genes or proteins involved in cuproptosis or possible pathway for the occurrence of cuproptosis requires further investigation. Furthermore, *in vitro* cell validation and animal experiments are needed to confirm the precise roles of these genes or proteins in cuproptosis and diabetes. Subsequently, large-scale human gene analysis should be necessarily conducted to determine their specific roles in the development of diabetes. These validation efforts require significant time, resources, and expertise. Even if the relevance of cuproptosis to diabetes has been confirmed, the development of related drugs still faces many challenges and difficulties. Besides, the action mechanisms of these genes and proteins should still be further understood to forge the foundation for the development of novel therapeutic strategies for diabetes. Therefore, despite some progress, significant work remains to be done in this field, holding considerable potential.

## Author contributions

JQ: Writing – original draft. YW: Writing – review & editing. QW: Writing – review & editing.
